# Safety and efficacy of *Momordica charantia* Linnaeus in pre-diabetes and type 2 diabetes mellitus patients: a systematic review and meta-analysis protocol

**DOI:** 10.1186/s13643-018-0847-x

**Published:** 2018-11-15

**Authors:** Emanuel L. Peter, Serawit Deyno, Andrew Mtewa, Félicien Mushagalusa Kasali, Prakash B. Nagendrappa, Duncan Sesaazi, Casim Umba Tolo, Patrick Engeu Ogwang

**Affiliations:** 1 Pharm-BioTechnology and Traditional Medicine Center, World Bank-Africa Center of Excellence (ACE II), Mbarara University of Science and Technology, P.O. Box 1410, Mbarara, Uganda; 2 Department of Innovation, Technology Transfer & Commercialization, National Institute for Medical Research, 2448 Baracka Obama drive, P.O. Box 9653, Dar Es Salaam, Tanzania; 3 Department of Pharmacology, School of Medicine, College of Medicine and Health Sciences, Hawassa University, Awasa, Ethiopia; 4 Chemistry Section, Department of Applied Sciences, Malawi Institute of Technology, Malawi University of Science & Technology, Limbe, Malawi; 5 Department of Pharmacy, Faculty of Medicine and Pharmacy, Official University of Bukavu, Bukavu, Democratic Republic of Congo; 6 School of Integrative Health Sciences, Trans-disciplinary University, Bengaluru, India

**Keywords:** NIDDM, Bitter gourd, Systematic review, Protocol, Meta-analysis

## Abstract

**Background:**

*Momordica charantia* Linnaeus (Cucurbitaceae) has been used traditionally as a nutritious food and as a herbal medicine for type 2 diabetes mellitus. However, human studies that investigated its glycemic control have generated inconsistent findings. Therefore, this systematic review and meta-analysis is aimed at evaluating the safety and efficacy of *M. charantia* L. preparations in human studies that have investigated its role in glycemic control.

**Methods:**

This protocol has been prepared according to Preferred Reporting Items for Systematic Review and Meta-Analysis Protocols (PRISMA-P). The review will include randomized clinical trials and non-randomized clinical trials. The included studies will have assessed glycemic control of *M. charantia* preparations with placebo or standard oral anti-hyperglycemic agents in adult pre-diabetes and/or type 2 diabetes mellitus patients and have at least 4 weeks of follow-up. The primary outcomes of review are fasting blood glucose levels, glycosylated hemoglobin A1c, and post-prandial blood glucose level. Electronic database search for published literatures will be conducted without language restriction in EMBASE, MEDLINE/PubMed, the Cochrane Library, SCOPUS, Web of Sciences, and CINAHL databases. Search for gray literatures and references of the retrieved full-text articles will be conducted in Google, Google Scholar, OpenGrey, ProQuest dissertations & Theses, British Library EThos, and university digital library systems. Two independent reviewers will later evaluate full texts, extract data, and assess risk of bias of eligible articles. Publication biases will be assessed by testing asymmetry of funnel plot using Egger’s or Begg’s tests while heterogeneity will be assessed using Cochran *Q* test, *P* value, and *I*^2^. Revman software version 5.3 will be used for meta-analysis including subgroup and sensitivity analysis.

**Discussion:**

This systematic review and meta-analysis will investigate both safety and efficacy of *M. charantia* preparations in type 2 diabetes mellitus. The review results will be published in a peer-reviewed journal. The results will bring better understanding of clinical outcomes in treatment of type 2 diabetes mellitus patients and highlight gaps for future research.

**Systematic review registration:**

PROSPERO CRD42018083653.

**Electronic supplementary material:**

The online version of this article (10.1186/s13643-018-0847-x) contains supplementary material, which is available to authorized users.

## Background

Diabetes mellitus is a growing global public health problem and an important cause of morbidity, disability and mortality. In 2017, an estimated 425 million adults globally had been diagnosed with diabetes mellitus [1]. The low- and middle-income countries have in recent years witnessed a dramatic increase in the prevalence of diabetes mellitus predominantly due to increased population aging, obesity, physical inactivity, and poor quality diet [2]. The international diabetes federation has projected that number of diabetes mellitus cases would increase to 629 million by 2045 if no serious preventive measures are immediately taken [1]. The most common form of diabetes mellitus is type 2 diabetes mellitus (T2DM) which accounts for about 90% of diabetes mellitus cases. It is predominantly due to failure of the bodily tissues to respond to insulin or synthesize enough insulin [3].

Treatment of T2DM involved the use of oral anti-hyperglycemic agents (OHAs) alone or combined in order to achieve optimal glycemic control [4, 5]. The American Diabetes Association (ADA) has been recommending OHAs and guidelines for adopting new advances in treatment with a major focus on improving management of patients with T2DM [6–8]. Although the advances in treatment options have subsequently contributed to improved glycemic control, they are often expensive and associated with a number of adverse effects [9, 10].

In line with the above, herb-based therapies and dietary supplements have become alternative to mainstream medical treatment. Unlike allopathic medicines, herbal medicines are used in their natural form, have few adverse effects and readily accessible to majority of patients [11, 12]. Studies in different countries have estimated 30–76% of patients with T2DM use herbal medicines [13, 14]. These medicines offer great potential for management of T2DM through provision of safe and effective antidiabetic drugs [15].

*Momordica charantia* Linnaeus (Family Cucurbitaceae) is the most studied herb for its anti-hyperglycemic effect in vivo and in clinical studies [16, 17]. It is a tropical and subtropical vine, widely common in Brazil, Asia, and some parts of east Africa including Tanzania [18], and it has been commonly used as nutritional food and medicine for centuries. Bioactive compounds such as charantin, vicine, p-insulin, momordicoside S, momordicoside T, conjugated linolenic acid, linoleic acid, and conjugated linoleic acid were isolated from whole fruit, seed, and pulp have anti-hyperglycemic activity with diverse mode of actions [19–21]. The diversity of actions [22, 23], has drawn significant attention from researchers in the field of drug discovery and resulting surge in clinical studies investigated its anti-hyperglycemic effects in T2DM. However, such studies have produced inconsistent findings in relation to clinical efficacy [17], and no systematic documentation of adverse effects has been undertaken [16]. Hence, the need for this systematic review and meta-analysis is to systematically review and synthesize evidences on the safety and efficacy of *M. charantia* as an alternative to conventional OHAs for the glycemic control in patients with type 2 diabetes mellitus. Specifically, this systematic review and meta-analysis is designed to answer the following questions: (1) What is the efficacy of *M. charantia* preparations in controlling plasma glucose level? (2) Does *M. charantia* Linn preparations safe when used by adult patients with type 2 diabetes mellitus? (3) Does duration of consumption and dosage of *M. charantia* preparations influence its safety and/or efficacy?

## Methods

**Fig. 1:**
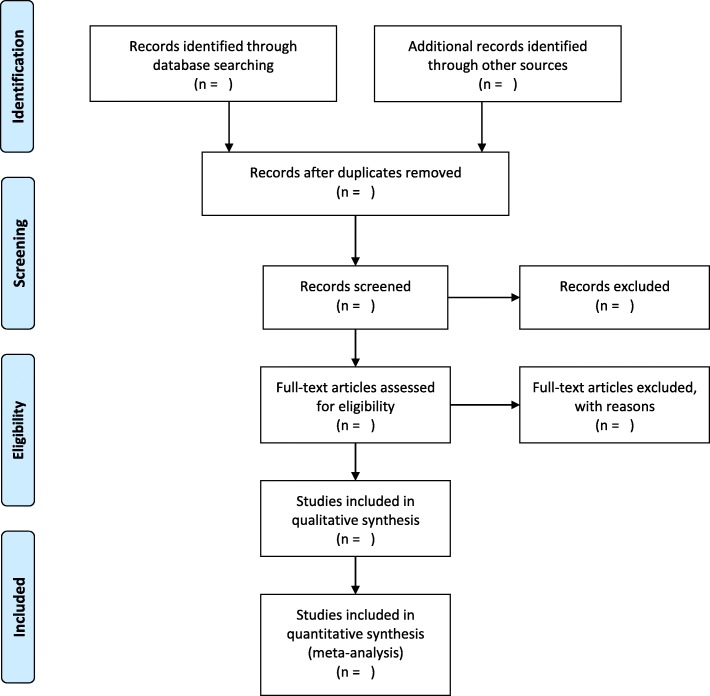
PRISMA flow diagram for study inclusion

This systematic review will be conducted in accordance to the Preferred Reporting Items for Systematic Review and Meta-Analysis (PRISMA) guidelines [24]. We will use PRISMA flow diagram to show articles selection and screening (Fig. 1). This protocol is developed according to the Preferred Reporting Items for Systematic Review and Meta-Analysis Protocol (PRISMA-P). PRISMA checklist was used to ensure the quality of the protocol as shown in Additional file 1 [25]. Our protocol has been registered in the international prospective register of systematic review with registration number PROSPERO CRD42018083653. Any amendments to the current registered protocol will be submitted to the PROSPERO database along with the reasons for such changes. The amended version of the protocol will then be made public through the database.

### Eligibility criteria

#### Inclusion criteria

Randomized controlled trials (RCTs) and non-randomized clinical trials will be included in the systematic review. The systematic review and meta-analysis will consider studies conducted in adult patients aged 18 years and older who have been diagnosed with pre-diabetes or type 2 diabetes mellitus of either sex from any country. The criteria used for diagnosis of pre-diabetes and T2DM should fit the standard diagnostic criteria of the World Health Organization (WHO) and/or the American diabetes associations (ADA) [7, 26–29]. Follow-up time of the included study should be at least 4 weeks for both primary and secondary outcomes, since clinical studies have indicated that this is the minimum period of time required for treatments to produce meaningful changes in glucose control as assessed by glycosylated hemoglobin A1c (HbA1c) concentrations [30].

#### Exclusion criteria

Pre-post studies with no comparison groups and conference abstract without adequate data will not be included. We will also exclude studies conducted on patients with concomitant endocrinopathies affecting their blood glucose levels such as T2DM with hypothyroidism.

### Search strategy for primary studies

A three-step search strategy will be adopted. Initially, we will search EMBASE, MEDLINE/PubMed, SCOPUS, CINAHL, Web of Science, and the Cochrane library using the keywords: *Momordica charantia*, bitter gourd, bitter melon, type 2 diabetes, NIDDM, clinical trial, and adult and human studies. The key words will be combined using boolenic logic terms “AND,” “OR,” and “NOT.” Then, analysis of the text words contained in the title and abstract and of the index terms used to describe article will be done. A second search using all identified keywords and index terms will be undertaken across all included databases. Thirdly, the reference list of all identified reports and articles after full-text screening will be searched for additional studies. No language restriction will be imposed during search and identification of studies. The key words which will be used in this study are included in Table 1.

**Table 1:** Search strategies for CINAHL database

No.	Query
S31	S13 AND S22 AND S30
S30	S23 OR S24 OR S25 OR S26 OR S27 OR S28 OR S29
S29	AB prospective stud*
S28	TI prospective stud*
S27	AB human stud*
S26	TI human stud*
S25	AB Clinical trial*
S24	TI Clinical trial*
S23	(MH “Clinical Trials+”)
S22	S14 OR S15 OR S16 OR S17 OR S18 OR S19 OR S20 OR S21
S21	AB non-insulin dependent diabetes mellitus
S20	TI non-insulin dependent diabetes mellitus
S19	AB “NIDDM”
S18	TI “NIDDM”
S17	“NIDDM”
S16	AB type 2 diabetes mellitus
S15	TI type 2 diabetes mellitus
S14	(MH “Diabetes Mellitus, Type 2”)
S13	S1 OR S2 OR S3 OR S4 OR S5 OR S6 OR S7 OR S8 OR S9 OR S10 OR S11 OR S12
S12	AB bitter melon
S11	TI bitter melon
S10	(MH “Melon”)
S9	AB “bitter gourd”
S8	TI “bitter gourd”
S7	“bitter gourd”
S6	AB “momordica”
S5	TI “momordica”
S4	“momordica”
S3	AB ““*Momordica charantia*””
S2	TI ““momordica charantia””
S1	““momordica charantia””

#### Searching for unpublished literature

Gray literature will also be included in this systematic review and meta-analysis. The search in Google scholar, Google, OpenGrey, ProQuest dissertations & Theses, British Library EThos, and university digital library systems will be done for conference papers, technical reports, thesis, and dissertations. To obtain data from ongoing clinical trials or unpublished trials, we will search through https://www.clinicaltrials.gov and contact research teams directly.

#### Search time interval

Since earlier clinical trials were reported in 1960s–1970s [31, 32], studies published from 1 January 1960 to 30 April 2018 will be considered for inclusion in this systematic review and meta-analysis to capture all possible clinical trials.

### Study dates

The systematic review and meta-analysis has been started in November 2017 and expected to end on 30 April 2018.

### Study records

#### Data management

Syntax for each database will be generated and set for a weekly update alert. In PubMed, an NCBI (National Center for Biotechnology Information) account will be created and password shared among reviewers. All identified articles will be pooled into Mendeley software, and later citations will be imported into Covidence an online systematic review platform accessible via https://www.covidence.org. The Covidence online software will handle duplicates, screen titles, and abstracts; carry out full-text screen of the uploaded PDF files; and do data extraction.

#### Selection process

Two independent reviewers (ELP and SD) will conduct the title and abstract screening in duplicate to identify eligible articles using predefined criteria. Full text of the eligible articles will be obtained and assessed against the inclusion and exclusion criteria. Any disagreements between the reviewers during full-text assessment will be resolved by discussion and consensus, when no resolution is reached, a third reviewer (AM) will be involved in a decision. Inter-rater agreement screening will be estimated using kappa statistic [33]. Further clarification would be sought from the study authors as deemed necessary to determine eligibility and obtain additional data.

#### Data collection process

Data from included studies in the systematic review and meta-analysis will be extracted using a standardized data extraction tool from Joanna Briggs institute meta-analysis of statistics assessment and review instrument (JBI-MAStARI) as shown in Additional file 2. The data extracted will include specific details about the interventions, population, study methods, and outcomes of interest to the review objective.

### Data items

#### Intervention

Mono or polyherbal preparation of whole fruit, seeds, or pulp of *M. charantia* in any dose and dosage was administered orally, alone, or in combination with oral anti-hyperglycemic agents.

#### Comparators

Placebo and standard oral anti-hyperglycemic agents or nutritional preparations were used as comparators.

### Outcome measures

#### Primary outcomes

- Fasting plasma glucose levels (FPG)
- Glycosylated hemoglobin A1c (HbA1c)
- Post-prandial plasma glucose level (PPG)

### Secondary outcomes

- Serum cholesterol
- Body weight
- Body mass index
- Serum creatinine level
- Alanine aminotransferase (ALT)
- Aspartate transaminase (AST)
- Incidence and severity of adverse effects

### Risk of bias in individual studies

Selected papers will be assessed by two independent reviewers (ELP and SD) for methodological validity prior to inclusion in the review. The included randomized trials will be assessed by using standardized Cochrane risk of bias tool [34]. The bias will be graded as low, high, or unclear. While non-randomized studies will be assessed using “risk of bias in non-randomized studies - of interventions” ROBINS-I [35]. In non-randomized studies, biases due to severity of diseases, comorbidities, and number of concomitant medications are anticipated, hence will initially be evaluated, after which additional unpredicted biases will be determined for each individual study and disagreements between the two primary reviewers will be resolved through discussion with a third reviewer (AM). In addition, reviewers will evaluate the authenticity of gray literature with the help of checklist developed specifically for gray literature by Jess Tyndall at Flinders University (AACODS) [36].

### Data synthesis

Qualitative syntheses will be done prior to meta-analysis of the included studies. In qualitative syntheses, a narrative approach will be employed in which summary of findings from multiple studies will be explained primarily in textual form. Where appropriate, the narrative syntheses will involve manipulation of statistical data in which findings will be presented in form of tables and figures to established patterns and variations.

Quantitative data will be pooled in statistical meta-analysis using RevMan software 5.3 (Cochrane collaboration). Simple analysis of final values (SAFV) analytical method for continuous variables will be employed. In this method, estimate of effect of intervention will be calculated simply as a difference in means of FPG, PPG, and HbA1c at follow-up between groups. The inverse of variance-weighted method will be used for pooling the weighted mean differences and its 95% confidence intervals [37]. For categorical data, effect sizes will be expressed as odds ratio. Subgroup analysis will be done by study design, duration of treatment, dose, and nature of formulation, i.e., either *M. charantia* alone or in combined with other herbs. An attempt will be made to explore sensitivity analysis by restricting methodological features such as quality restriction, study type, and design restriction.

#### Heterogeneity assessment

The Cochran *Q* test and its *P* value will be used to evaluate heterogeneity between primary studies where as *I*^2^ statistic will be used for assessing heterogeneity severity. The heterogeneity severity will be evaluated before performing the analysis to decide whether to use random effects model or fixed effect model [38]. Factors that affect the heterogeneity which include primary study quality score and design, dosage amount, and nature of formulation, i.e., mono herbal and poly herbal therapy will be assessed.

### Meta-bias

#### Publication bias assessment

Publication bias will be assessed by testing asymmetry of funnel plot using Egger’s test or Begg’s tests [39, 40]. The test for funnel plot asymmetry will not be used when there are fewer than ten primary studies in the meta-analysis because test power is generally too low to distinguish chance from real asymmetry [41]. If publication bias is significant, trim and fill method will be used for correcting the probable publication bias. In addition, the significant asymmetry of funnel plot will be interpreted in the context of susceptibility to other biases that might explain it.

### Confidence in cumulative evidence

The GRADE system will be used for assessing quality of the evidence of each outcome using eight criteria which are indirectness, inconsistency, imprecision, and publication bias in addition to four criteria of risk of bias assessment tool [42]. Using GRADEpro/GDT, an online software programme accessible through https://gradepro.org/, quality of cumulative evidence for each outcome will be graded as high, moderate, low, or very low in a summary of findings table.

## Discussion

Treatment of type 2 diabetes mellitus involved multipronged approaches to mitigate both macro and micro vascular complications. Previous studies have established diverse mechanism of action through which *M. charantia* exerts its effects [23, 24]. These unique features of the plant have attracted attention of both researchers in the field of drug discovery and patients with type 2 diabetes mellitus. The situation has resulted into large number of scientific publications which disseminate a wide array of information ranging from ethno-pharmacological findings, pre-clinical studies, formulation studies, and clinical validation of various *M. charantia* preparations. The first systematic review was published in August 2012 [16]. In this work, only four randomized controlled trials (RCT) designs were included. The risk of bias assessment of these studies was generally high and therefore authors could not have established sufficient evidence on the effects of *M. charantia* preparations on type 2 diabetes mellitus. Contrary to previous work, our systematic review will, in addition to RCT design, include non-randomized clinical studies that evaluated *M. charantia* with placebo or standard oral anti-hyperglycemic agents. This could increase number of studies included and therefore improve strength of evidence. Similar inconclusive evidence was reported in another systematic review [17]. In their work, only three RCTs that compared the effect of *M. charantia* with placebo were included in a meta-analysis. The authors neither explained the search strategy used for obtaining unpublished data nor included unpublished reports in the systematic review. Limiting the search to only published reports could have biased the estimation of safety and efficacy in the review [43]. The fact that only three clinical trials were included in the analysis also weakens the ability to obtain meaningful quantitative estimate in meta-analysis. The use of arbitration of a third independent individual for methodological validation and control of bias strengthens the objective position that the findings of this systematic review and meta-analysis will have.

## Additional files

Additional file 1: PRISMA-P 2015 Checklist. (DOCX 32 kb)

Additional file 2: Standardized data extraction tool modified from JBI-MAStARI. (DOCX 15 kb)

## Abbreviations

ACE: Africa Centre of Excellence
ADA: American Diabetes Association
ALT: Alanine aminotransferase
AST: Aspartate aminotransferase
DM: Diabetes mellitus
FPG: Fasting plasma glucose
HbA1c: Glycosylated hemoglobin A1c
NIDDM: Non-insulin-dependent diabetes mellitus
OHAs: Oral hypoglycemic agents/oral anti-hyperglycemic agents
PPG: Post-prandial glucose
PRISMA: Preferred Reporting Items for Systematic Review and Meta-Analysis
PRISMA-P: Preferred Reporting Items for Systematic Review and Meta-Analysis Protocols
RCTs: Randomized controlled trials
T2DM: Type 2 diabetes mellitus
WHO: World Health Organization
