# Supplementary material for: Safety and efficacy of Momordica charantia Linnaeus in pre-diabetes and type 2 diabetes mellitus patients: a systematic review and meta-analysis protocol
Source: Syst Rev. 2018 Nov 15;7:192. doi: 10.1186/s13643-018-0847-x (PMC6238397; doi:10.1186/s13643-018-0847-x)
Supplement: Supplementary file 2 — Standardized data extraction tool modified from JBI-MAStARI. (DOCX 15 kb) [file 13643_2018_847_MOESM2_ESM.docx]

**Additional file 2: Standardized data extraction tool modified from JBI-MAStARI**

Reviewer……………………………………………………. Date………………………….

Author………………………………………………………. Year………………………….

Journal………………………………………………………Record No…………………….

**Study Method**

RCT Quasi-RCT Longitudinal/Prospective

Retrospective

**Participants**

Setting………………………………………………….……………………………………….

Population……………………………………………………….…………………………….

**Sample size**

Group A(*M.Charantia*)…………………………Group B (Control)…………………………..

**Interventions**

Intervention A (*M.Charantia*)…..……………………………………………………………..

Intervention B (Control……….……………………………………………………………….

Author conclusion

………………………………………………………………………………………………………………………………………………………………………………………………………………

Reviewer conclusion

……………………………………………………………………………………………………………………………………………………………………………………………….

**Dichotomous/Categorical data**

| Outcome | Intervention –*M. Charantia*  Number/total No. | Intervention-Control  Number/total No. |
| --- | --- | --- |
|  |  |  |
|  |  |  |
|  |  |  |
